# Functional outcome and complication rate after percutaneous suture of fresh Achilles tendon ruptures with the Dresden instrument

**DOI:** 10.1186/s10195-018-0511-1

**Published:** 2018-09-18

**Authors:** Sebastian Manegold, Serafim Tsitsilonis, Jakob Schumann, Tobias Gehlen, Alison N. Agres, Johannes Keller, Markus Gesslein, Florian Wichlas

**Affiliations:** 1 Center for Musculoskeletal Surgery, Charité—University Medicine Berlin, Campus Virchow Clinic, Augustenburger Platz 1, 13353 Berlin, Germany; 2 Julius Wolff Institute, Charité—University Medicine Berlin, Augustenburger Platz 1, 13353 Berlin, Germany; 3 Clinic for Orthopaedics and Traumatology, Klinikum Nürnberg Süd, Paracelsus University, Breslauer Str. 201, 90471 Nuremberg, Germany; 4 Clinic for Orthopaedics and Traumatology, University of Salzburg, Müllner Hauptstraße 48, Salzburg, Austria

**Keywords:** Achilles tendon, Rupture, Percutaneous, Minimal invasive

## Abstract

**Background:**

The aim of this study was to evaluate the outcome of patients with a rupture of the Achilles tendon (ATR) treated percutaneously with the Dresden instrument in the hands of surgeons others than its inventors.

**Materials and methods:**

118 patients (FU rate: 77.1%) with an acute ATR treated with the Dresden instrument were retrospectively evaluated. The following data were evaluated: pain intensity, functional limitation, Hannover score, Achilles tendon total rupture score (ATRS), AOFAS ankle-hindfoot score, Tegner activity score, complications, maximum calf circumference (MCC) on both sides, and the Matles test for tendon lengthening. The effect of the time point of the surgery after trauma was examined.

**Results:**

Hannover scores and ATRSs were good; AOFAS scores were excellent. Almost all patients returned to sporting activities postoperatively, and 66.1% were able to return to their previous level. The Tegner activity score revealed a slight posttraumatic decrease (*p* = 0.009) in the level of physical activity overall (pre-injury: 5.37 ± 0.15; postoperatively: 4.77 ± 0.15). The re-rupture rate was 2%. No sural nerve lesions and no infections were reported. Even after 3 years, there was still a difference in MCC that was correlated with inferior clinical score and AT lengthening. Patients treated within the first 2 days after ATR showed inferior clinical outcomes in terms of AOFAS score, ATRS, and functional limitations.

**Conclusions:**

Percutaneous ATR suture with the Dresden instrument is a safe and reliable method. Low complication and re-rupture rates, good clinical results, and a high rate of return to play support this fact. The time point of the operation may influence the outcome.

## Introduction

The Achilles tendon (AT) is the thickest and strongest tendon in the human body, with acute AT ruptures accounting for about 35% of all tendon tears [1]. The rise in the participation of the general population in sporting activities observed over the last 30 years has led to a tenfold increase in the incidence of AT rupture (ATR) to 21 per 100,000 over that period [2, 3]. The majority of these ruptures occur in male patients between 30 and 40 years old during recreational sports [4], although there has been an increase in the rate of ATR in the age group between 40 and 59 years old over the last decade [5]. Rupture of the AT is a potentially severe injury requiring long-term rehabilitation and involving high socioeconomic costs. In professional sports, it can be a career-ending injury [6], while it can also affect the everyday life of the nonprofessional athlete. Several treatment options are now available, spanning from conservative therapy to open surgical reconstruction. Identifying the appropriate treatment can be challenging given the scope of each technique and the relatively good functional results of all of the treatment options [7–9]. While open surgical treatment leads to lower re-rupture rates than conservative therapy (4% vs. 10%), with fewer days off work [7, 8, 10], it also results in a higher complication rate, with potentially disastrous results [8, 9]. Minimally invasive percutaneous techniques were developed as an alternative solution [11]. Despite the similarly low re-rupture rate and a lower wound complication rate compared to open surgery, percutaneous techniques have been only cautiously accepted, as they were previously notorious for sural nerve lesions [12–15]. In an effort to overcome this problem, Amlang et al. developed a suture device (the Dresden instrument) and a surgical technique where the key steps are performed subfascially, thus theoretically minimizing the risk of sural nerve injury [16]. The first studies reported very good results with very low complication rates. Nonetheless, relatively few studies support the use of this percutaneous technique for the treatment of acute ATR. The aim of the present study was to independently evaluate the postoperative outcomes of patients treated with the Dresden instrument in the hands of surgeons others than its inventors, with a minimum follow-up period of 1 year.

## Materials and methods

For the needs of the present study, all patients with an acute (< 10 days) ATR that was operatively treated in our institute with a percutaneous suture using the Dresden instrument over a 7-year period (10/2003–10/2010) were included and retrospectively evaluated. All the patients gave their informed consent prior to inclusion in the study; the local ethics committee approved the study (EA2/095/11). An electronic ICD-9 search was conducted and the identified patients were contacted by telephone or by mail to invite them for a follow-up examination. Overall, 153 patients were identified and contacted. In total, 118 patients (follow-up rate: 77.1%) agreed to take part in the study. The median patient age of the included patients was 42 years (range 24–73 years). The majority of the patients were men (*n* = 102; 84.3% male). The mean follow-up was 33.45 months (range 12–82 months; SD 21.67). In most cases (89.2%), the ATR happened due to sports-related injuries. The Achilles tendon rupture was located on the left in 53.7% and on the right side in 46.3%. Indications for percutaneous suture were type 2a and 3a ruptures (according to the Amlang classification) that were no older than 10 days, with sonographically incomplete adaptation of the ruptured ends of the tendon in 20° plantar flexion [16]. Percutaneous suture was not performed for older or open ruptures, or in re-rupture cases. The detailed surgical technique employed has been described elsewhere [17]. Briefly, a 2-cm skin incision was made about 5 cm proximal to the rupture site. The Dresden instrument was inserted subfascially while ensuring that the underlying paratenon remained intact. Both instruments were advanced until they were as close to the tendon insertion as possible, with one lying medial and one lateral to the distal tendon stump. Two straight needles armed with resorbable PDS II No.1 (Ethicon^®^, Johnson & Johnson) were pierced through the skin–instrument–tendon–instrument–skin. The instruments were then pulled back so that the sutures were diverted through the proximal incision, and the pull-out strength of the sutures was tested thoroughly. The repair was completed with a Krackow locking stitch in the proximal tendon stump, while the ATR was secured in an overtightened manner. Postoperatively, the foot was placed in a walker in the 30° equinus position for a period of 6 weeks with partial weight-bearing. After the sixth week, the heel height was reduced 10° per week and weight-bearing was increased as tolerated. From the ninth postoperative week, the patients were allowed to walk in a normal shoe. A standardized physiotherapeutic regimen was handed out to each patient and consisted of functional muscle training, proprioceptive training, and stretching exercises, while scar tissue massage was started from the third postoperative week. The following data were included in the analysis: subjective pain intensity and subjective functional limitation were measured on a 10-point visual analogue scale (VAS); the Hannover score [18]; the Achilles tendon total rupture score (ATRS) [19]; the AOFAS ankle-hindfoot score; and the Tegner activity score for assessing the pre-injury and postoperative levels of sporting activity. Peri- and postoperative complications were evaluated. Moreover, the maximum calf circumference (MCC) at a level of 15 cm distally to the medial knee joint line and the ankle range of motion (ROM) of both the injured and the contralateral sides were documented, and the Matles test was carried out in order to examine tendon lengthening. A single trained person performed all measurements with an intraobserver variability of less than 5%. Finally, we also examined the effect of the time point of surgery after trauma.

### Statistical analysis

Continuous variables were expressed as mean ± standard deviation (SD) values, and categorical variables as percentages (%). The Kolmogorov–Smirnov test was used to assess distribution normality. For parametric variables, the paired Student’s *t* test was used to compare two groups; for nonparametric variables, the Wilcoxon signed-rank test was implemented. For categorical variables, differences were assessed with the *χ*^2^ test or Fisher’s exact test. Correlations were examined with either the Pearson product-moment correlation coefficient or Spearman’s rank correlation coefficient. Differences were considered to be statistically significant if *p* < 0.05.

## Results

### Clinical outcome scores

The clinical outcome scores showed good results. The mean VAS score for pain was 0.6 points (range 0–7; SD 1 point). The vast majority of the patients (81.4%) reported minimal pain intensity of between 0 and 1. Only one patient (7.0 points) reported pain of >3 points on the VAS. The VAS score for subjective functional limitation was slightly higher—the mean VAS score was 1.3 points (range 0–8; SD 1.4 points). Almost half of the patients (44%) reported a functional limitation of up to 4.0 points on the VAS. Only two patients, both with a pathological Matles test, reported a subjective functional limitation of 8.0 on the VAS (Table 1). The results for the Hannover score and the ATRS—which are validated for Achilles tendon ruptures—were good, while the AOFAS scores were rated as excellent (Table 1). The patient’s sex did not influence the outcome scores.

**Table 1:** Clinical outcomes for the total study population showed good to excellent scores overall as well as low levels of pain intensity and functional limitation

Score	Mean ± SD	Median	Min	Max
Hannover score	85.2 ± 12.1	88	25	100
ATRS	85.4 ± 14.8	90	19	100
AOFAS score	95.3 ± 6.6	100	74	100
VAS score for functional limitation	1.4 ± 1.5	1.0	0	8
VAS score for pain	0.6 ± 1.0	1	0	7

*ATRS* Achilles tendon total rupture score, *AOFAS* American Orthopaedic Foot & Ankle Society, *VAS* visual analogue scale

### Level of sporting activity and return to sports

Almost all patients were able to return to sporting activity (93.3% of patients participated before ATR and 91.3% postoperatively), with 66.1% of them able to return to their previous level of sporting activity. However, almost one-quarter of the patients (28 patients; 23.7%) changed the type of sport they played, while 12 patients (10.2%) quit sports. The Tegner activity score revealed a slight, though statistically significant, posttraumatic decrease (*p* = 0.009) in the level of physical activity (pre-injury level: 5.37 ± 0.15; postoperative level: 4.77 ± 0.15). Most patients that changed sports swapped from sporting activities with quick changes of direction to jogging or other less demanding sports. The reasons for reduced sporting activity were heterogeneous: fear of a re-rupture was the most important factor, followed by a subjective loss of strength as well as changes in recreational priorities. Unsurprisingly, the results for the Hannover score (91 pts. vs. 71 pts.), the VAS for functional limitation (1.0 vs. 1.95), and the AOFAS score (100 pts. vs. 87 pts.) were significantly worse in the patients that quit sports (*p* < 0.0001). Differences in ATRS were not statistically significant (92 pts. vs. 80 pts.; *p* = 0.128).

### Complications

No intra- or perioperative complications were observed. Among the 153 patients who were initially contacted, a re-rupture of the AT was observed in 3 patients (2%); all of these re-ruptures occurred during the first 16 weeks postoperatively and underwent revision surgery for open AT repair. No lesions of the sural nerve and no infections were reported.

### Maximal muscle circumference, ROM, and tendon length

**Fig. 1:**
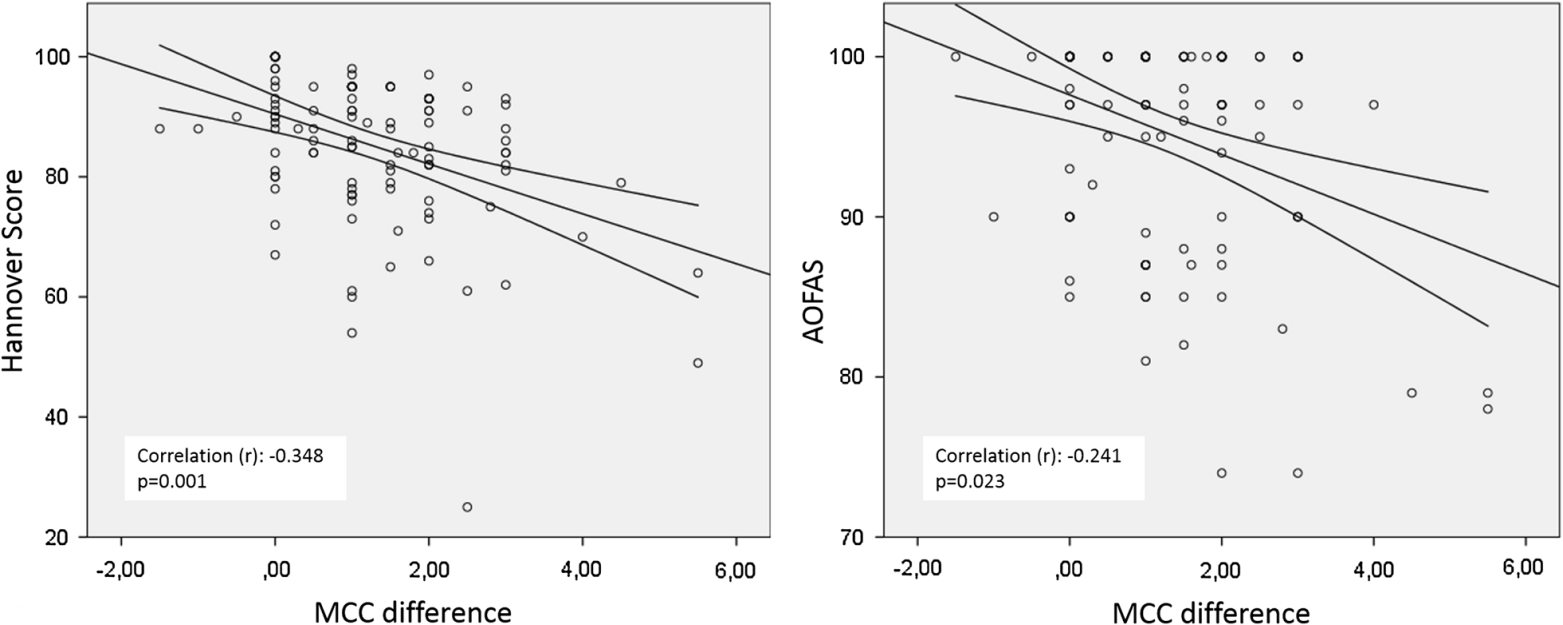
Side-to-side difference in maximum calf circumference (MCC). Patients with larger side-to-side differences in MCC had lower clinical outcome scores. There were negative correlations between the side-to-side difference in MCC and clinical outcome scores. *AOFAS* American Orthopaedic Foot & Ankle Society, *ATRS* Achilles tendon total rupture score, *VAS FL* visual analogue scale score for functional limitation. Level of significance: *p < 0.05

Even after almost 3 years postoperatively, a difference in MCC was still present. Although the mean difference was small (1.2 cm), the MCC was significantly lower (*p* = 0.001) on the injured side than for the contralateral calf (Table 2). Significant negative correlations were observed between MCC and clinical outcome scores (Fig. 1). There were negative correlations of the side-to-side difference in MCC with the Hannover score as well as the AOFAS score. In terms of ankle ROM, the mean plantar flexion (injured: 46.1 ± 8.6; intact: 47.9 ± 8.2°) and mean dorsal extension (injured: 14.5 ± 4.6°; intact: 14.9 ± 4.9°) showed no significant difference between the injured and the intact sides. The Matles test, which detects side-to-side differences in Achilles tendon length, showed no secondary lengthening or shortening of the Achilles tendon in comparison to the healthy side in 77% of the patients (91/118 patients). Patients with AT lengthening presented significantly inferior Hannover scores (76 pts. vs. 88 pts; *p* < 0.0001) and had significantly more dorsal extension (*p* < 0.0001) than those with equal tendon lengths.

**Table 2:** Maximum calf circumference as a parameter of muscle atrophy: a significant reduction in maximum calf circumference on the injured side was still present at the mid-term follow-up

Calf circumference (cm)	Mean ± SD	Median	Minimum	Maximum
Injured	36.4 ± 2.9	43.0	27.0	43.0
Contralateral	37.6 ± 2.6	45.5	30.0	45.5
Difference	1.2 ± 1.2	1.0	0	5.5
Significance	*p* = 0.001*			

Level of significance: *p < 0.05

### Time point of surgery

Patients that were operated on within the first 2 days after Achilles tendon rupture (*n* = 31) showed inferior clinical outcomes in terms of AOFAS score, ATRS, and VAS score for functional limitation when compared to those who were operated on later than 48 h after trauma (*n* = 87) (Table 3).

**Table 3:** Dependence of clinical outcomes on the interval between Achilles tendon rupture (ATR) and surgery (patients who underwent surgical Achilles tendon repair within the first 2 days after trauma showed inferior clinical outcomes in terms of ATRS, Hannover score, and VAS scores for pain intensity and functional limitation compared to those who were operated on later than 48 h after trauma)

Interval between ATR and surgery (days)	AOFAS score	Hannover score	ATRS	VAS FL	VAS pain
0–2	94 ± 7	80.6 ± 13.8	78.8 ± 16.3	2.0 ± 1.6	0.91 ± 1.28
3–8	96 ± 6.4	86 ± 11.5	87 ± 13.8	1.1 ± 1.2	0.49 ± 0.78
Significance	0.063	0.039*	0.028*	0.004*	0.046*

*AOFAS* American Orthopaedic Foot & Ankle Society, *ATRS* Achilles tendon total rupture score, *VAS FL* visual analogue scale score for functional limitation, *VAS pain* visual analogue scale score for pain

Level of significance: *p < 0.05

## Discussion

The present study shows that the treatment of acute ATR with a percutaneous suture technique using the Dresden instrument delivers good clinical results combined with very low re-rupture and surgical complication rates. To our knowledge, the present study includes one of the largest ATR patient populations to be treated with the Dresden instrument [16, 20–22]. The clinical scores were very good in the present study. Similar results were noted in the studies of ATR patients treated with the Dresden instrument reported by Amlang et al. and Keller et al. [16, 20]. Additionally, two other studies have reported low complication rates and good functional results after treatment with the Dresden instrument [21, 22]. Treatment with the Achillon^®^ device (Integra, Plainsboro, NJ, USA) also seemed to deliver similar results [23, 24]. All of the evaluated scores were significantly correlated with patient satisfaction. Analyzing the specific scores for ATR enables further differentiation of the results. In the present population, 11 patients showed inferior Hannover scores, despite the fact that the AOFAS scores were very good in four of those patients and good in six more. This discrepancy in score outcomes reveals a specific issue with clinical studies of Achilles tendon ruptures: Achilles-tendon-specific problems, such as one-leg stand limitations, decreased jumping ability, and muscle atrophy are not taken into consideration in the commonly used AOFAS score, and seem to be better reflected in specific scores such as the ATRS and the Hannover score. Based on our findings, we would recommend the use of such Achilles-tendon-specific scores and not the general AOFAS score to evaluate outcomes after ATR. Indeed, the ATRS and the Hannover score were specifically developed for Achilles tendon ruptures [18, 19]. Despite this fact, the ATRS and (especially) the Hannover score have not found consistent use in the literature for the long-term follow-up of patients who have undergone percutaneous suture with the Dresden instrument. This means that there are no comparable score data for this percutaneous suture technique. However, the ATRS and Hannover scores noted in our study were comparable to those seen in other studies of different minimally invasive techniques [10, 25, 26]. Achilles tendon ruptures mainly occur in stop-and-go sports, and one of the major goals of treatment is a return to play at the pre-injury level. In comparable studies by Amlang et al. [16] and Keller et al. [20], the proportion of the patients who achieved a return to play at the same level after percutaneous suture with the Dresden instrument ranged from 51 to 80%. Those data were supported by our study, as 66% of the patients in our study achieved the same level in their sporting activity after rehabilitation. In a meta-analysis of 85 studies encompassing over 5500 patients, Zellers et al. noted that 18–100% of patients were able to achieve their pre-injury levels in their favored sporting activities after percutaneous suture with the Dresden instrument [27]. This wide percentage range should, however, be interpreted with care, as there is neither a standardized definition nor an evaluation protocol for a return to play. Nevertheless, they show that while functional deficits do occur after ATR, some patients seem to overcome these deficits and achieve full rehabilitation. The factors that influence functional rehabilitation are presently unclear. Recent work suggests that functional deficits after ATR are caused by structural impairments of the muscle–tendon unit due to altered tendon properties [28]. However, to unravel the mystery of functional deficits after ATR, more research must be done in the field of biomechanics to comprehend the process of tendon healing and the factors that influence it. In the present population, the re-rupture rate of the Achilles tendon was 2%, while no sural nerve lesions or infections were observed. Amlang et al. also reported a re-rupture rate of 3.2%, no lesions of the sural nerve, and only one late superficial wound infection after tendon healing [29]. In the study of Keller et al., a re-rupture rate of 2% was reported, while no nerve lesion or wound infection was described [20]. These data are comparable to the complication rate of a similar minimally invasive technique: the Achillon device. A meta-analysis of that technique which included 253 patients reported a re-rupture rate of 3.2% and an infection rate of 0.8% [30]. Pooling these data, minimally invasive techniques such as the Dresden instrument or Achillon device seem to have surmounted the issue with the wound complication rate of open procedures in AT repair, as well as the problem of sural nerve lesions. A meta-analysis by Del Buono and coworkers underlines these findings [15]. In their study, they presented an overall surgical complication rate (deep infection, wound necrosis, scar tissue adhesion) of 8% (30 of 375 patients) versus 0.25% for minimally invasive techniques (1 of 406 patients). Furthermore, the re-rupture rate after minimally invasive AT repair was 2.2%, as low as the re-rupture rate after an open approach (3.5%) [15].

While low re-rupture rates and surgical complication rates are prerequisites for a reliable surgical technique, full motor function and a quick return to play at the pre-injury level are the predominant goals for recreational and professional athletes after ATR. However, it remains unclear which therapy—conservative or operative—is superior and the most suitable for the individual patient to achieve these goals. Although good clinical results have been reported for both operative and nonoperative treatments [9, 15, 31, 32], long-lasting functional deficits are exhibited after ATR regardless of therapy, such as a reduction in plantar flexion strength, a reduced range of motion, or limited plantar flexion moment due to increased tendon stiffness [10, 33–36]. One important aspect seems to be the resulting muscle atrophy, which eventually leads to a loss of strength and inferior functional results [37–39]. A reduction in MCC was observed on the injured side in the present population in comparison to the healthy side. The importance of this finding is reflected in the correlation between MCC and inferior clinical scores in our study. The atrophy of the calf on the injured side seemed to persist even 33 months after the operation in the present study, and it is known that other tendons such as the supraspinatus muscle in the shoulder do not completely recover over time after rupture [10, 40]. The observed difference in calf circumference was equally distributed across different age groups; no correlation with age was seen. This finding indicates that even younger patients with a potentially higher capacity for muscle growth are failing to achieve a full muscular recovery. However, differences in maximum calf circumference cannot necessarily be equated with muscle atrophy. MRI studies are needed to further differentiate between muscle atrophy and fatty degeneration. Patients with a pathologic Matles test presented larger differences in MCC between sides than those with restored AT length (according to the Matles test) did. However, this finding was not statistical significant.

Based on the results of our study, one outcome-influencing factor could be timing of surgery: patients who were operated on later than 2 days after ATR scored significantly better than those who underwent immediate surgery within the first 48 h after trauma. As different phases of tendon healing are associated with different phases of inflammatory response [41], an altered expression pattern of inflammatory markers might be one possible explanation. To our knowledge, this is the first study to report such data. Unfortunately, we could not find a correlation between increasing preoperative time interval (ATR to surgery) and improved clinical outcome nor determine an optimal point of time for the operation. Until this finding is confirmed by other studies, these results must be interpreted with care, but they should prompt further studies on different phases of tendon healing.

### Limitations

Care should be taken when interpreting the postoperative level of activity and the maximum calf circumference. Due to the retrospective nature of the study, a recall bias in the pre-injury activity level is inevitable, especially after a follow-up period of up to 7 years. There are several potential reasons why ATR patients perform recreational sports to a lower level after surgery. Moreover, assuming that the maximum calf circumference on the contralateral side is equivalent to that on the injured side at the time of AT rupture is a critical but commonly used method in retrospective studies. Prospective data collection would have avoided this aspect. Finally, force measurements and ultrasound to determine AT length were not included in our study. Including those methods would have led to more precise conclusions about the functional and structural impairment of the muscle–tendon unit.

The present study shows that percutaneous suture of an Achilles tendon rupture with the Dresden instrument is a safe and reliable method of treating this injury. Low complication and re-rupture rates, good clinical results, and a high rate of return to play support this fact. Muscle atrophy, measured as muscle circumference, and tendon lengthening resulted in an inferior functional outcome. Future studies on the exact roles of outcome-influencing parameters such as muscle atrophy, tendon properties, and the time point of the operation are needed to elucidate the reasons for functional impairment after this injury.
